# Risk factors for lower limb lymphedema after gynecological cancer treatment: a systematic review

**DOI:** 10.3389/fonc.2025.1561836

**Published:** 2025-05-20

**Authors:** Tina Decorte, Marie Cerckel, George Bou Kheir, Chris Monten, Katrien Vandecasteele, Luc Vanden Bossche, Nele S. Pauwels, Caren Randon

**Affiliations:** ^1^ Department of Physical Medicine and Rehabilitation and Clinic for Lymphatic Disorders, Ghent University Hospital, Ghent, Belgium; ^2^ Faculty of Medicine and Health Sciences, Ghent University, Ghent, Belgium; ^3^ Department of Urology, Ghent University Hospital, Ghent, Belgium; ^4^ Department of Radiation Oncology and Clinic for Lymphatic Disorders, Ghent University Hospital, Ghent, Belgium; ^5^ Department of Radiation Oncology, Ghent University Hospital, Ghent, Belgium; ^6^ Department of Physical Medicine and Rehabilitation Ghent University Hospital, Ghent, Belgium; ^7^ Knowledge Centre for Health Ghent, Ghent University and Ghent University Hospital, Ghent, Belgium; ^8^ Department of Thoracic and Vascular Surgery and Clinic for Lymphatic Disorders, Ghent University Hospital, Ghent, Belgium

**Keywords:** gynecological cancer, risk factors, lower extremity lymphedema, review, incidence

## Abstract

**Introduction:**

As treatments in gynecological cancer improve, the number of cancer survivors increases, with more patients facing long-term side effects of their treatment. One debilitating side effect is lower limb lymphedema (LLL). Unlike upper limb lymphedema (ULL), diagnosis of LLL remains challenging due to the absence of a clear definition, bilateral presentation complicating comparison, and confusion with post-operative weight changes. This systematic review investigated incidences and risk factors for LLL.

**Methods:**

We systematically searched PubMed, Embase, and CENTRAL databases for articles on LLL following treatment for pelvic gynecological cancer from 1979 to November 2024. Two independent researchers extracted data, based on predefined inclusion and exclusion criteria. We assessed bias using the Risk of Bias in Non-Randomized Studies (ROBINS-I) tool and adhered to PRISMA reporting guidelines.

**Results:**

Our review included 46 studies, with incidence rates varying widely across cancer types: 7.4-55.9% in cervical cancer, 1.2-47% in endometrial cancer, 5.6-30.4% in ovarian cancer, and 10.1-43% in vulvar cancer. Several risk factors for LLL emerged. Notably, lymphadenectomy, the number of removed lymph nodes, radiotherapy, and a body mass index (BMI) exceeding 25 kg/m² were significant risk factors. Surgical technique did not impact LLL risk.

**Conclusion:**

LLL frequently occurs following gynecological cancer treatments, emphasizing the importance of careful monitoring and proactive management in clinical settings. Overall, the findings highlight the complexity and variability in risk factors for LLL across different gynecological cancers. The significant heterogeneity in study designs, populations, and methodologies underscores the need for standardized approaches in future research to better understand and mitigate the risk of LLL in these patients.

**Systematic review registration:**

https://www.crd.york.ac.uk/PROSPERO, identifier CRD42020198642.

## 1 Introduction

Gynecological cancers affect the uterus, ovaries, cervix, vulva, and vagina. The incidence of these cancers is approximately 11% in the United States and 17% worldwide (1). Treatment of those cancers often damage the lymphatic drainage due to removal of the lymphatic nodes or fibrosis following radiotherapy or systemic treatment (2). This may cause secondary LLL, a chronic and irreversible condition consisting of lymph fluid accumulation in the lower limbs or genital region. Lymphadenectomy (LA) and radiotherapy are associated with a significant risk of developing LLL. Fibrosis of the lymph nodes (LN) following radiotherapy can lead to lymphatic insufficiency and inadequate lymphatic transport (3). Obstruction of the lymphatic system results in lymphatic congestion and increased pressure in the lymphatic channels (4). Subcutaneous fluid accumulation triggers discomfort such as swelling of the limb, which can be unilateral or bilateral, a feeling of heaviness or numbness, skin changes or sometimes even pain. Indirectly it can lead to psychological or social distress, affecting the ability of these women to function in their daily activities with an additional negative impact on the patients’ quality of life (QoL) (4, 5). This review focuses on the risk factors for developing LLL after cancer treatment. Does the number of lymph nodes removed, the surgical technique used, whether in combination with radiotherapy and or chemotherapy have more impact on the development of LLL? What is the role of patient-related factors such as obesity? The development of LLL after cancer treatment is influenced by a combination of patient-related, cancer-related, and treatment-related factors. Identifying these risk factors may lead to strategies for early detection of LLL and better orientation of preventive measures. Providing accurate information about the risks of procedures is crucial for patient education and informed decision-making regarding treatment options.

Previous reviews, such as those by Biglia et al., Dessources et al., and Hu et al., have explored various aspects of LLL in gynecological cancer patients (6–8). Biglia et al. highlighted the significant impact of lymphadenectomy and radiotherapy on LLL development (6). Dessources et al. focused on the pathophysiology, incidence, and risk factors associated with LLL, emphasizing the need for more prospective studies and objective metrics (7). Hu et al. provided a comprehensive analysis of predictive factors and developed a model for identifying patients at risk of LLL following lymphadenectomy (8). This review aims to build on these findings by providing a systematic analysis of the risk factors for LLL, considering the combined effects of different treatment modalities and patient-related factors.

## 2 Methods

This systematic review and meta-analysis were conducted according to the *Cochrane Handbook for Systematic Reviews of Interventions* (version 5.1.0) (9) and reported complying with the Preferred Reporting Items for Systematic Reviews and Meta-Analyses (PRISMA) statement (10). The review was registered on PROSPERO, December 2020 (CRD42020198642). We performed a search of PubMed, Embase and CENTRAL databases on LLL after gynecological cancer treatment to retrieve articles from 1979 up to 01 November 2024. The search strategies are provided in Appendix 1. Additionally, the reference lists of included studies were searched manually. To identify ongoing studies, ClinicalTrials.gov and the ISRCTN registry were also searched. Inclusion criteria were original studies of women aged 18 years and older with gynecological cancer treated with surgery and/or radiotherapy, where the primary outcome measure was risk factors for LLL in relation to the incidence of LLL. Exclusion criteria were review articles and meta-analyses, articles not published in English, inaccessible full-text articles, case reports and studies with less than 15 patients. Relevant data from the eligible full texts were extracted by two authors (TD and MC) independently using pre-structured data sheets in the software program DistillerSR. The data sheets were pre-designed to extract data on risk factors in relation to LLL after gynecological cancer. Disagreements were resolved by discussion and consensus with a third researcher (CR).

We conducted an independent assessment of the risk of bias using the Risk of Bias in Non-Randomized Studies (ROBINS-I) tool. The tool covers 7 domains through which bias can occur. The first two domains deal with issues before the start of the interventions being compared (“baseline”), and the third domain deals with the classification of the interventions themselves. The other four domains address issues after the start of the interventions (11).

All analyses were performed using RStudio version 2023.06.1 Build 524 with the meta package (version 6.3-0) and the metafor package (version 4.6-0) for meta-analysis and meta-regression, respectively. A random-effects model was applied using the restricted maximum likelihood (REML) method to account for heterogeneity among studies. Odds ratios and 95 percent confidence intervals were computed, while forest plots were generated to visually display study results, including effect sizes, confidence intervals, sample sizes, and weights. Meta-regression was performed to explore the relationship between the odds ratio and the mean number of lymph nodes dissected. The regression line, along with the 95 percent confidence interval, was plotted against the mean number of lymph nodes to evaluate potential moderating effects.

## 3 Results

**Figure 1:**
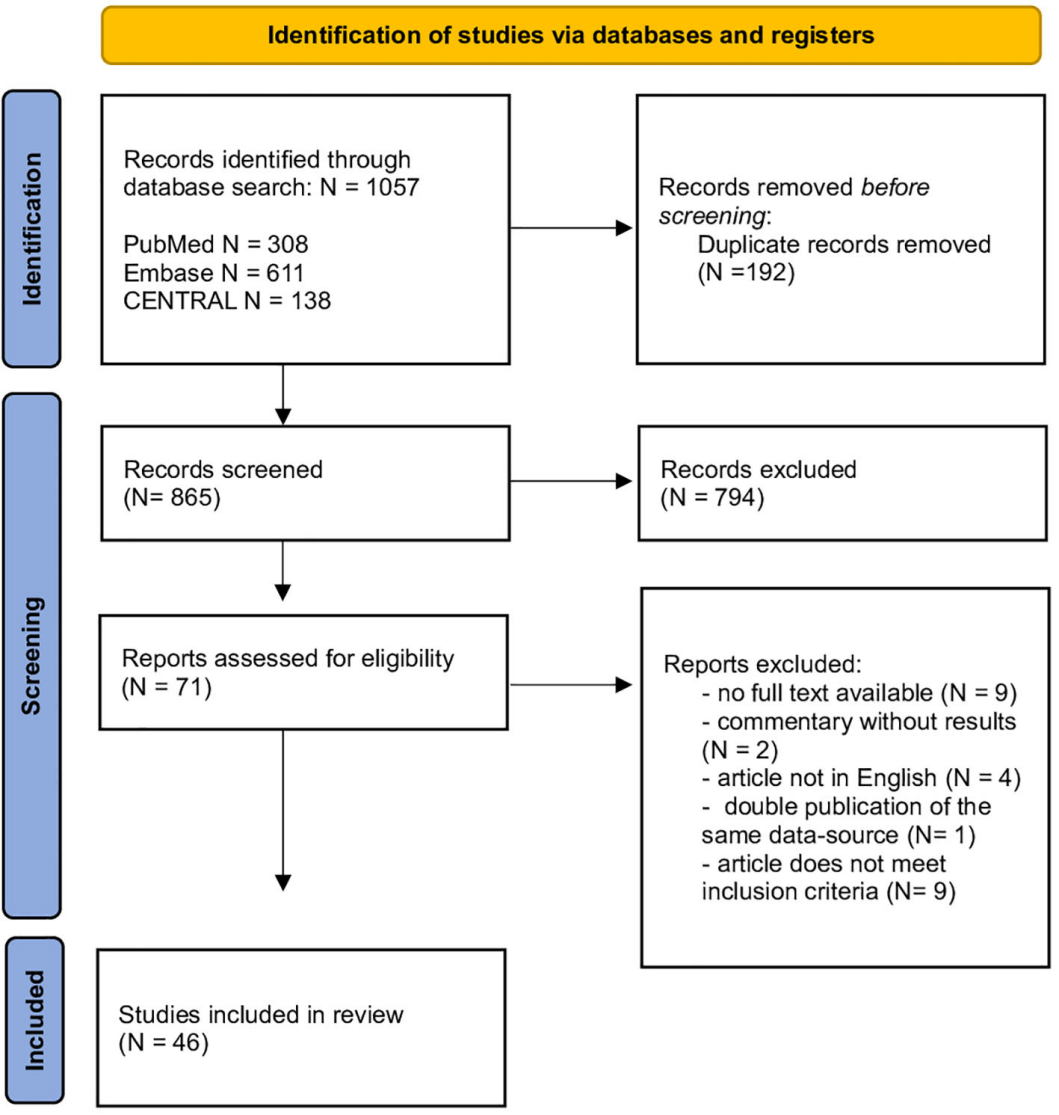
Prisma flowchart of the screening process.

Our search yielded 1057 records of which 865 were retained after removing duplicates. After title and abstract review, 71 articles remained eligible and were reviewed for inclusion and exclusion criteria. This resulted in the inclusion of 46 studies. **Figure 1** shows a flowchart of the literature screening process.

### 3.1 Characteristics of the included studies

Most studies investigating risk factors following gynecological cancer treatment have been retrospective in nature, with only 6 prospective studies published to date. Two of these prospective studies, conducted by Wedin et al., included 235 patients with endometrial cancer (12, 13). The sample sizes in the studies varied significantly, ranging from 25 up to 19.027 patients. **Table 1** summarizes the basic characteristics of the studies included in the review for LLL after cancer treatment.

**Table 1:** Basic characteristics of the included studies.

Study, (ref)	Year	Country	Study design	Number of patients (n)	Methods for diagnosis of LLL	Cancer type	Risk factors evaluated
Casarin et al. (14)	2024	Italy	Prospective cohort study	239	Lymphedema Questionnaire	Endometrial	Age Charlson Comorbidity Index Type of surgery FIGO stage Adjuvant treatment
Bruce et al. (15)	2023	USA	Retrospective cohort study	201	Clinical	Vulvar	Radiotherapy
Lee et al. (16)	2022	Korea	Retrospective cohort study	858	Clinical Perimetry Lymphoscinitgraphy	Cervical + Endometrial	Age BMI Type of hysterectomy LN removed Radiotherapy Chemotherapy
Liu et al. (17)	2022	China	Case-control study	253 (case group) + 506 (control group)	Clinical GCLQ Perimetry Limb Volume	Cervical	BMI Co-morbidities Radiotherapy Chemo + radiotherapy Level of education
Cibula et al. (18)	2021	Europe	Prospective cohort study	150	Clinical	Cervical	No significant risk factors
Lee et al. (19)	2021	Korea	Retrospective cohort study	19.027	Clinical	Endometrial	Age Radiotherapy Chemotherapy Multimodal treatment
Wedin et al. (12)	2021	Sweden	Prospective longitudinal study	235	Clinical LYMQOL Perimetry Limb Volume	Endometrial	Age BMI Cardiac disease, Diabetes Diuretics Type of surgery Number LN dissected + Location Radiotherapy
Wong et al. (20)	2021	UK	Retrospective cohort study Cross-sectional study	376	Clinical GCLQ	Cervical, Endometrial, Ovarian, Vulvar	Type of cancer: Vulvar
Carlson et al. (21)	2020	USA	Prospective cohort study	914	Perimetry Limb Volume	Cervical (138), Endometrial (734), Vulvar (42)	No significant risk factors
Kunitake et al. (22)	2020	Japan	Retrospective cohort study	356	Clinical	Cervical (121), Endometrial (151), Ovarian (84)	Type of cancer Tumor stage
Pigot et al. (23)	2020	Australia	Prospective cohort study	235	Self-Reported leg swelling (SRLS) Limb Volume Bioimpedance spectroscopy (BIS)	Endometrial	BMI Co-morbidities Number of LN dissected Chemotherapy
Rebegea et al. (24)	2020	Romania	Retrospective cohort study	326	Clinical Limb Volume	Cervical (186) + Endometrial (140)	Obesity Number of LN dissected (cervical) Number of LN with metastasis (cervical)
Togami et al. (25)	2020	Japan	Retrospective cohort study	289	Clinical	Endometrial	CIN Removal
Togami et al. (26)	2020	Japan	Retrospective cohort study	167	Clinical	Cervical	No significant risk factors
Wedin et al. (13)	2020	Sweden	Prospective longitudanl study	235	Clinical LYMQOL Perimetry Limb Volume	Endometrial	Not investigated
Yoshihara et al. (27)	2020	Japan	Retrospective cohort study	711	Clinical	Cervical, Endometrial and Ovarian	Age > 50 years Radiotherapy
Chang et al. (28)	2019	Korea	Retrospective cohort study	228	Clinical Questionnaire Lymphoscintigraphy Perimetry Limb Volume	Cervical, Endometrial	Type of Surgery Number of LN dissected Radiotherapy
Volpi et al. (29)	2019	Italy	Retrospective cohort study	249	Clinical	Endometrial	BMI Tumor grade Diabetes, Hypertension, Metabolic syndrome Type of surgery Removal of CINDEIN Radiotherapy Chemotherapy Positive LN
Togami et al. (30)	2018	Japan	Retrospective cohort study	169	Clinical	Cervical	Number of LN dissected CIN removal
Hayes et al. (31)	2017	Australia	Prospective cohort study	408	Clinical BIS	Endometrial (171), Ovarian (72), Cervical (28), Vulvar (15)	Age Type of cancer Number of LN dissected Radiotherapy
Kim et al. (32)	2017	Korea	Retrospective cohort study	511	Clinical	Cervical (155), Uterine (114), Ovarian (133), Vulva (3)	BMI Type of cancer Number of LN dissected Radiotherapy
Kuroda et al. (33)	2017	Japan	Retrospective cohort study	264	Clinical Questionnaire	Cervical (78), Endometrial (113), Tube (2), Ovary (68), Vaginal (3)	BMI Number of LN dissected Radiotherapy
Bae et al. (34)	2016	Korea	Retrospective cohort study	274	GCLQ	Endometrial	Number of LN dissected Radiotherapy Chemo + radiotherapy
Ki et al. (35)	2016	Korea	Retrospective cohort study	413	Lymphoscinitgraphy Limb Volume	Ovarian	Tumor stage PLND + PALND
Mitra et al. (36)	2016	USA	Retrospective cohort study	212	Clinical Questionnaire	Endometrial	Co-morbidities Number of LN dissected LN possitivity
Beesley et al. (37)	2015	Australia	Retrospective cohort study	643	Questionnaire	Endometrial	Age Number of LN dissected Chemotherapy NSAID
Berger et al. (38)	2015	USA	Retrospective cohort study	146	Clinical	Vulvar	No significant risk factors
Biglia et al. (39)	2015	Switzerland	Cross-sectional study	152	Questionnaire	Cervical (34), Endometrial (95), Ovarian (23)	No significant risk factors
Cirik et al. (40)	2015	Turkey	Retrospective cohort study	99		Vulvar	BMI Number of LN dissected LN involvement
Hareyama et al. (41)	2015	Japan	Retrospective cohort study	358	Clinical	Cervical (100), Endometrial (121), Ovarian (137)	Number of LN dissected
Todo et al. (42)	2015	Japan	Retrospective cohort study	318	Clinical	Uterine corpus	Number of LN dissected Removal of CINDEIN Radiotherapy
Yamazaki et al. (43)	2015	Japan	Retrospective cohort study	398	Clinical	Cervical	Radiotherapy
Deura et al. (44)	2014	Japan	Retrospective cohort study	126	Clinical Limb Volume	Cervical (42), Endometrial (43), Ovarian (39), Other (2)	Age Chemo + radiotherapy
Hoogendam et al. (45)	2014	Netherlands	Retrospective cohort study	100	Clinical	Cervical	No significant risk factors
Yost et al. (46)	2014	USA	Retrospective observational study	591	Lower Extremity Lymphedema Screening Questionaire (LELSQ)	Endometrial	BMI Lymphadenectomy Radiotherapy
Achouri et al. (47)	2013	France	Retrospective cohort study	88	Clinical	Cervical (17), Endometrial (35), Ovarian (36)	No significant risk factors
Kondo et al. (48)	2013	Japan	Retrospective cohort study	321	Clinical	Cervical (129), Endometrial (119), Ovarian (70), Other (3)	PALND Location dissceted LN Persistent lymphocyst
Kim et al. (49)	2012	Korea	Retrospective cohort study	596	Clinical Limb Volume	Cervical	Radiotherapy
Kasuya et al. (50)	2011	Japan	Retrospective cohort study	228	Clinical	Uterine	Radiotherapy
Ohba et al. (51)	2011	Japan	Retrospective cohort study	155	Clinical	Cervical	Location dissected LN Radiotherapy
Walker et al. (52)	2011	UK	Retrospective cohort study	56	Clinical	Vulvar	Number of LN dissected
Todo et al. (53)	2010	Japan	Retrospective cohort study	286	Clinical Lymphoscintigraphy Limb Volume	Endometrial	Number of LN dissected Removal of CINDEIN Radiotherapy
Tada et al. (5)	2009	Japan	Retrospective cohort study	694	Clinical	Ovarian (135) + Uterine (258 cervical + 301 endometrial)	Radiotherapy
Füller et al. (54)	2008	Germany	Retrospective cohort study	192	Clinical	Cervical	Surgical approach Number of LN dissected
Abu-Rustum et al. (55)	2006	USA	Retrospective cohort study	1289	Clinical	Uterine	Number of LN dissected
Ryan et al. (56)	2003	Australia	Retrospective cohort study	743	Clinical Questionnaire	Vulvar	At risk with removal of LN and follow up Radiotherapy
Hong et al. (57)	2002	Taiwan	Retrospective cohort study	228	Clinical	Cervical	Age Radiotherapy

BIS, Bioimpedance spectroscopy; BMI, Body Mass Index; CIN, circumflex iliac node; CINDEIN, circumflex iliac nodes distal to the external iliac nodes; GCLQ, Gynecologic Cancer Lymphedema Questionnaire; LA, Lymphadenectomy; LELSQ, Lower-Extremity Lymphedema Screening Questionnaire; LN, Lymph Nodes; LYMQOL, Lymphedema Quality of Life Questionnaire; NSAID, Nonsteroidal anti-inflammatory drugs; PALA, Para-aortic lymphadenectomy; PALND, Para-Aortic Lymph Node Dissection; PLA, Pelvic Lymphadenectomy; PLND, Pelvic Lymph Node Dissection.

### 3.2 Risk factors for gynecological cancer related lower limb lymphedema

The diagnosis of LLL was most frequently based on clinical examination using the International Society of Lymphology (ISL) grading system or questionnaires. Risk factors for LLL differ across various studies and types of cancer.

#### 3.2.1 Endometrial cancer

Twenty-eight of the included studies investigated risk factors for LLL after endometrial cancer. The studies comprised 4 prospective cohort studies, 1 cross-sectional study, and the remainder were retrospective cohort studies. Fourteen studies focused exclusively on endometrial cancer, while the other studies in the review included various gynecological tumors. This heterogeneity complicates combining these studies into a single meta-analysis. Only the studies describing risk factors for lymphedema, limited to endometrial cancer, and applying odds ratio were considered for meta-analysis.

##### 3.2.1.1 Patient-related risk factors

Seven studies investigated age as a risk factor, with 2 indicating that patients aged 60–65 years or older were at higher risk, contrarily to Wedin et al., who suggested younger women are at higher risk (12, 19, 37). However, due to the heterogeneity in study design, population, and methodology, pooling age-data for meta-analysis was not possible.

Among the 14 studies included for meta-analysis, 10 examined BMI as a potential risk factor. There was considerable variability in defining BMI thresholds. Only 4 studies reported a significant effect of BMI on LLL. The odds ratios for different BMI categories varied significantly across studies. For example, Yost et al. reported higher odds ratios for higher BMI categories (OR 4.69 (95% CI: 2.71-8.13) for BMI of 40.0 or higher), while Beesley et al. showed lower odds ratios (OR 0.9 (95% CI: 0.6-1.6) for BMI 25.0-29.9 and OR 0.9 (95% CI: 0.5-1.6) BMI 30.0-39.9) (37, 46). Heterogeneity among studies prevents a consistent conclusion. The wide confidence intervals (95% CI) indicate uncertainty in the estimates, with some studies including the null value (odds ratio = 1). This suggests that the effect of BMI on LLL is not consistent across studies. Given the significant variation in odds ratios and confidence intervals, pooling BMI as a risk factor for LLL may not be appropriate.

Other patient-related risk factors such as co-morbidities and smoking were only investigated in 5 studies (13, 23, 29, 36, 46). These studies suggest that certain co-morbidities, such as chronic heart failure, diuretics, calcium-antagonists, and NSAIDs, may negatively influence lymphedema development.

##### 3.2.1.2 Treatment-related risk factors

**Figure 2:**
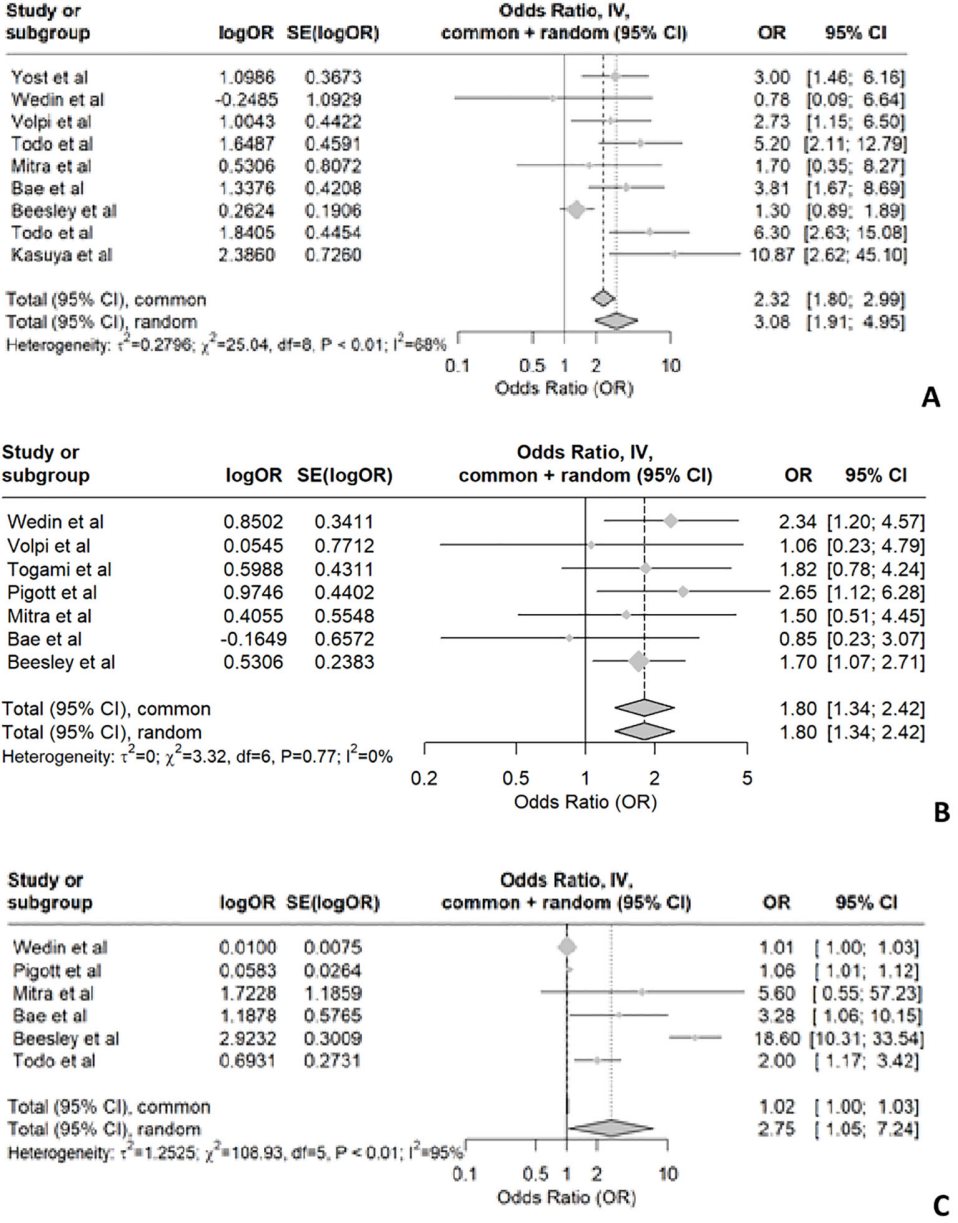
Shows the forest plot for radiotherapy **(A)**, chemotherapy **(B)** and number of lymph nodes dissected **(C)**.

Radiotherapy emerged as a significant risk factor in 9 studies, while 3 studies did not find any significance. The heterogeneity analysis for studies examining radiotherapy as a risk factor for LLL in endometrial cancer revealed significant variability among the included studies. The t² value was 0.2796, indicating the presence of between-study variance. The χ² test yielded a value of 25.04 with 8 degrees of freedom (df), and the associated p-value was less than 0.01, suggesting that the observed heterogeneity is statistically significant. Additionally, the I² statistic was 68%, indicating that 68% of the total variation across studies is due to heterogeneity rather than chance. Based on the statistical findings and the substantial heterogeneity observed, it is reasonable to conclude that radiotherapy is a risk factor for developing LLL among endometrial cancer patients. The odds ratios reported in the studies indicate an increased risk, and the significant between-study variance suggests that this effect is consistent across different studies, despite some variability (**Figure 2A**).

While some studies show a positive association between chemotherapy and LLL (12, 23, 37), others do not provide conclusive evidence. In patients with endometrial cancer, the pooled data suggest that chemotherapy is associated with the development of LLL, as indicated by the forest plot (**Figure 2B**) showing an OR of 1.8 with a 95% confidence interval of 1.34 to 2.42. This indicates statistically significant differences. However, the meta-analysis reveals no significant heterogeneity among the included studies, suggesting a high level of consistency. It is important to note that most studies did not specify whether patients received radiotherapy in addition to chemotherapy. Only two studies described chemotherapy alone as a risk factor. Due to the lack of subgroup analysis and differentiation between treatment groups (chemotherapy alone, chemotherapy and radiotherapy, and radiotherapy alone), the pooled data may not accurately reflect the impact of chemotherapy alone on the development of LLL.

Removal of circumflex iliac nodes (CIN) may contribute to the development of lymphedema in endometrial cancer according to two retrospective cohort studies involving 289 and 286 patients, respectively (25, 53). Most studies on endometrial cancer have consistently reported two significant risk factors for LLL: the number of lymph nodes removed and radiotherapy. However, there is no uniformity in the number of lymph nodes removed across studies. Among the 15 included studies in our analysis, 6 specifically investigated endometrial cancer and found a significant correlation between lymph node removal and risk of LLL (12, 23, 34, 36, 37, 42). The forest plot for the removal of lymph nodes shows a p-value of <0.01, indicating significant heterogeneity. The I² value is 95%, with T² = 1.2525, Chi² = 108.93, and df = 5. This suggests substantial variability in the effect sizes across the studies (**Figure 2C**).

**Figure 3:**
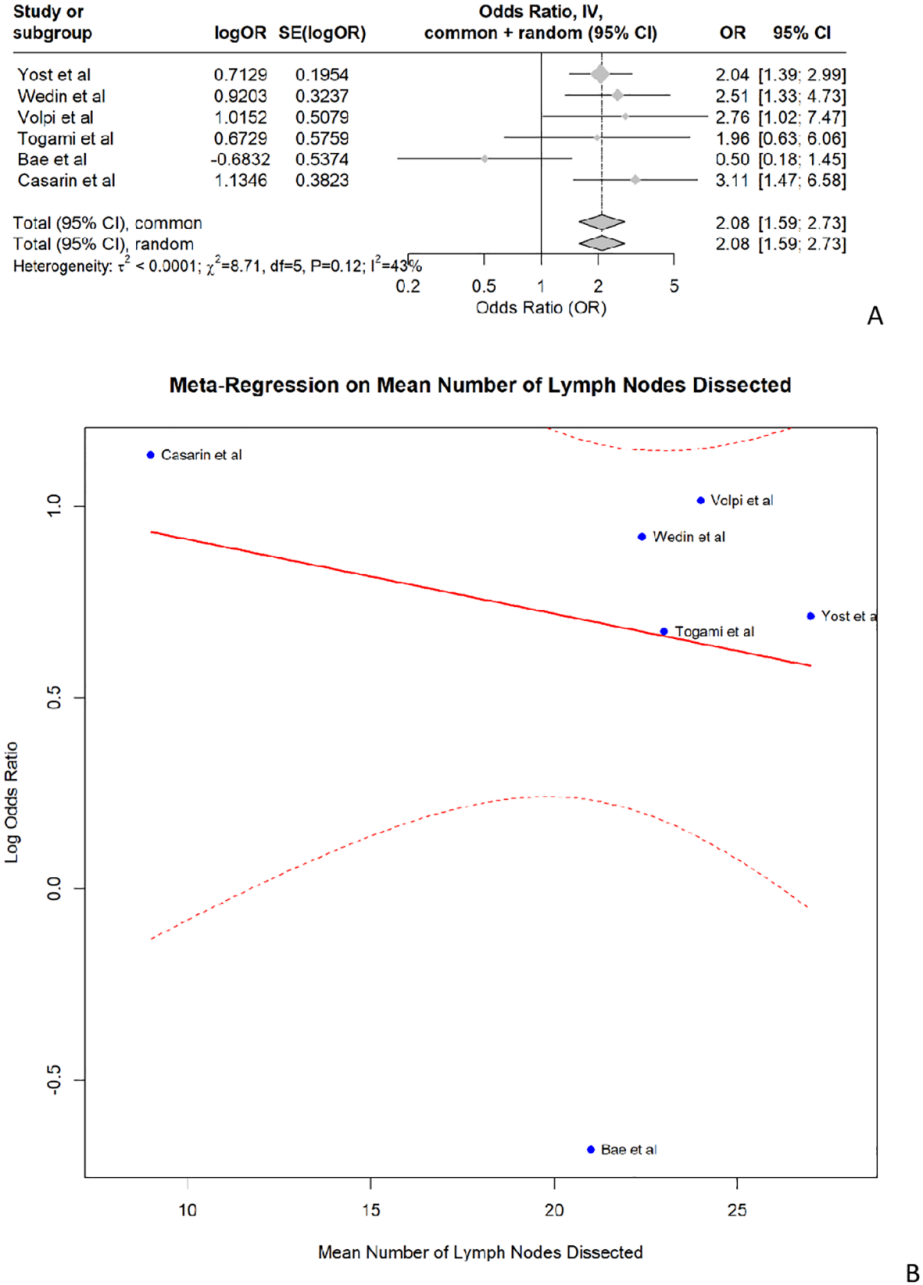
**(A)** shows the forest plot for lymphadenectomy and **(B)** shows the meta- regression of the median number of lymph nodes dissected.

Concerning the risk of LLL followingLA, the pooled odds ratio was 2.08 (95% CI: 1.59–2.73, p<0.001). The analysis showed moderate heterogeneity, with an I-squared value of 42.6%. The meta-analysis indicates that the studies are consistent and show no significant heterogeneity, with LA (compared to no lymph node dissection) being a significant risk factor for LLL in endometrial cancer patients. (**Figure 3A**) The meta-regression assessing the impact of the median number of lymph nodes dissected revealed no significant moderating effect, with an estimate of -0.0195 (p=0.622). Despite consistent findings that LA increases the risk of LLL, our meta-regression analysis did not show a significant dose-response relationship based on the number of lymph nodes retrieved. This suggests that factors such as variability in surgical technique, differing anatomical dissections (e.g., inclusion of circumflex iliac nodes), and patient-related variables may confound this relationship (**Figure 3B**).

No significant difference in LLL risk was found between surgical approach (open versus closed), application of retroperitoneal closure, type of hysterectomy: pelvic and para-aortic LA and pelvic LA alone (52.4% vs. 49.4%; p=0.630) (46). In a prospective study by Wedin et al. (12) they concluded that risk factors varied depending on the method of determining lymphedema. They found LA was a risk factor for LLL when assessed to BMI standardized volume, clinical grading, and patient-perceived swelling but not when evaluated by crude volume. **Table 2** summarizes the risk factors for LLL in endometrial cancer.

**Table 2:** Risk factors for LLL in endometrial cancer.

Risk factor	Number of studies	Findings	Comments
Age	7	Higher risk in patients aged 60–65 years or older; one study suggested younger women are at higher risk	Heterogeneity in study design and methodology prevented pooling age data for meta-analysis
BMI	10	Significant effect reported in 4 studies; odds ratios varied significantly across studies	Wide confidence intervals indicate uncertainty; pooling BMI data for meta-analysis may not be appropriate
Co-morbidities	5	Chronic heart failure, use of diuretics, calcium-antagonists, and NSAIDs may negatively influence LLL development	Limited number of studies; further research needed to clarify associations
Radiotherapy	9	Significant risk factor: heterogeneity analysis showed substantial variability among studies	Odds ratios indicate increased risk; significant between-study variance suggests consistent effect despite variability
Chemotherapy	3	Positive association in some studies; pooled data suggest statistically significant differences	Lack of subgroup analysis and differentiation between treatment groups may affect accuracy
Lymphadenectomy	6	Significant correlation between lymph node removal and risk of LLL; pooled odds ratio 2.08 (95% CI: 1.59–2.73, p<0.001)	Moderate heterogeneity: number of lymph nodes dissected does not significantly influence risk
Surgical Approach	1	No significant difference in LLL risk between open vs. closed approach, retroperitoneal closure, type of hysterectomy	Risk factors varied depending on method of determining LLL

The review of publications of LLL after treatment for endometrial cancer highlights several key risk factors. Age and BMI are significant patient-related factors, with older patients and those with higher BMI being at greater risk for developing LLL. However, the variability in defining BMI thresholds and the heterogeneity in study designs make it challenging to draw consistent conclusions. Radiotherapy is a significant treatment-related risk factor, with studies consistently showing an increased risk of LLL among patients who received radiotherapy. The interaction between lymph node removal and radiotherapy further complicates the risk profile, suggesting that patients undergoing extensive LA and radiotherapy are at the highest risk.

#### 3.2.2 Cervical cancer

The review included 11 studies that investigated risk factors for LLL in patients with cervical cancer. One prospective cohort study examined risk factors for LLL after cervical cancer treatment in 150 patients and found that the development of LLL was not impacted by the number of lymph nodes removed, surgical approach, age, BMI, or adjuvant radiotherapy. However, only 12% of the included patients received radiation therapy, which may have influenced this result. Another prospective study did not identify any significant risk factors for developing LLL (45).

##### 3.2.2.1 Patient-related risk factors

Four articles investigated whether BMI was an independent risk factor for developing LLL in patients with cervical cancer. Two of these articles did not find statistical significance (26, 49), while the other articles demonstrated a clear association, although they did not define the specific BMI values (17, 24). Co-morbidities as risk factors for LLL was investigated in only 1 article. Liu et al. found an increase in LLL risk if there was a history of coronary heart disease, vaginal disease, or abnormal menstruation (17). Interestingly, age does not appear to be a risk factor for cervical cancer according to 4 independent studies (18, 30, 43, 49). However, the study by Hong et al. suggested that age might be a risk factor for LLL in cervical cancer patients over 60 years old, although it was not significant for patients below 60 years old (57).

##### 3.2.2.2 Treatment-related risk factors

Multiple studies have explored whether the location of LN removal in cervical cancer is a risk factor. Notably, both circumflex iliac node (CIN) and circumflex iliac nodes distal to the external iliac nodes (CINDEIN) removal appear to be significant risk factors (26, 30, 43). Additionally, 1 study identified suprafemoral node removal as a potential risk factor (51).

**Figure 4:**
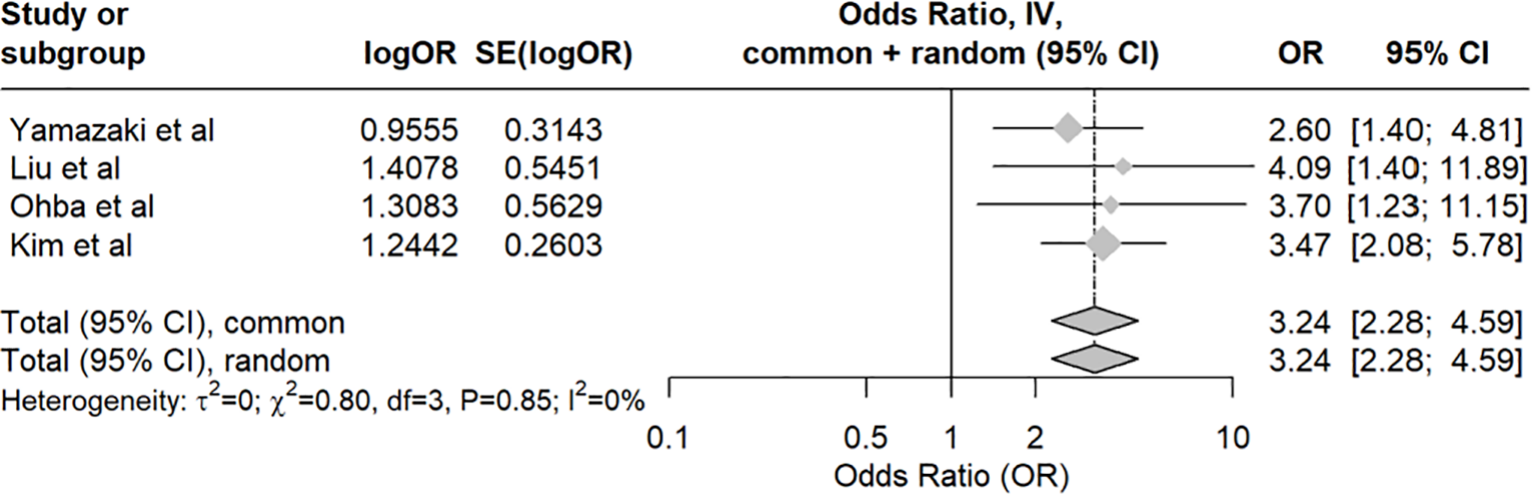
Shows the forest plot for radiotherapy.

In the 11 studies investigating cervical cancer, radiotherapy consistently appears as a significant risk factor for LLL in 5 studies (17, 43, 49, 51, 57), while 3 studies did not find a significant association (18, 30, 45). When combining the results of these studies, the examination of the association between radiotherapy and the development of LLL in cervical cancer patients shows no significant heterogeneity among the included studies (**Figure 4**). This indicates that the results are highly consistent across the studies. However, it is important to note that the lack of heterogeneity does not equate to statistical significance. The pooled data indicate that radiotherapy significantly contributes to the development of LLL in cervical cancer patients.

Three studies investigated chemotherapy as an independent risk factor, but none of them found any significant association (30, 45, 54).

#### 3.2.3 Ovarian cancer

In this review, only 2 studies specifically examined ovarian cancer to identify risk factors associated with the development of LLL. One study demonstrated an association between the number of LN removed and LLL, with an odds ratio of 1.025 (95% CI: 1.005–1.045) (35). Conversely, another study found that pelvic lymph node removal combined with radiotherapy was a risk factor for LLL in patients with ovarian or uterine cancer. No correlation was observed between surgical procedures and LLL (5).

#### 3.2.4 Vulvar cancer

Only 8 studies investigated risk factors in vulvar cancer, 7 were retrospective and 1was prospective (20, 21, 31, 38, 40, 52, 56). Out of the 8 studies, only 4 were unique to vulvar cancer, while the remaining 4 combined various gynecological cancers but included a separate analysis for vulvar cancer. One retrospective study of 146 patients with vulvar cancer found no associated risk factors in the development of LLL due to the low incidence rate of LLL in the study population (38). The prospective study including 42 patients with vulvar cancer also showed no significant risk factor (21). Four retrospective studies with respectively 56, 68, 15 and 99 patients, showed number of LN removed as a risk factor for LLL (31, 40, 52, 56).

#### 3.2.5 Gynecological cancer

Thirteen articles in the review described risk factors for developing LLL without distinguishing between the different types of gynecological cancer. The studies indicate varying levels of risk for developing LLL based on the different types of gynecological cancer and treatment factors, with some studies showing significant associations while others did not. This variability in findings, methodologies, and reported odds ratios complicates the analysis. Six of these articles specifically investigated whether the type of cancer influenced the development of edema. Yoshira et al. found in a study population of 711 patients that patients with cervical cancer had a greater risk of developing LLL compared to those with endometrial or ovarian cancer (OR: 1.912, p=0.001) (27). Similarly, Wong et al. reported a higher risk of LLL in patients with vulvar cancer compared to those with cervical, ovarian, and endometrial cancers, with a prevalence of 30.2% in vulvar cancer patients (OR: 11.0, CI: 3.2-38.3) (20). In contrast, studies by Kuroda et al., Kunitake et al., Hareyama et al., and Hayes et al. found no significant influence of the type of cancer on the development of LLL (22, 31, 33, 41). Additionally, differences in sample sizes, treatment modalities, and specific risk factors further complicate the ability to draw consistent conclusions across studies. Regarding patient-related risk factors, some studies identified younger age, higher BMI, and specific co-morbidities (eg. cellulitis) as significant for developing LLL (27, 33, 41). However, these results are inconsistent across studies, with some reporting no significant risk factors at all. Given the variability in findings, methodologies, and reported outcomes, conducting a meta-analysis would be challenging. The heterogeneity in the data, including differences in patient populations, risk factors assessed, and statistical methods used, makes it impractical to combine results into a single relevant meta-analysis.

### 3.3 Incidence of LLL in the included studies

**Figure 5:**
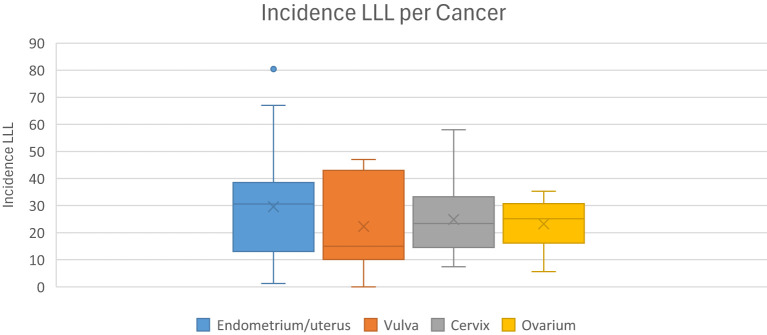
Incidence of LLL by cancer type.

The incidence of LLL varies widely in the literature, ranging from 1-49% and depends on the tumor type (58, 59). This variation may be due to the lack of a gold standard for diagnosis of LLL. In most literature, the diagnosis is mainly based on subjective symptoms. To further explore the impact of diagnostic methods on the reported incidence rates, we calculated the incidence of LLL for each included study based on the diagnostic method used. This analysis highlights how variations in diagnostic approaches, such as the use of questionnaires and clinical control, may influence the reported incidence rates. Our study showed incidence rates of 7.8-55.9% for cervical cancer, 1.2-47% for endometrial cancer, 5.6-30.4% for ovarian cancer and 10.1-43% for vulvar/vaginal cancer. The incidence of LLL by cancer type is shown in **Figure 5**.

### 3.4 Methodological quality

Bias due to confounding was classified as critical/serious in 8/46 studies where LLL was assessed in groups that were not comparable or with different follow-up periods between groups, or where mild cases of LLL were excluded due to lack of diagnostic tools for diagnosis of LLL. Most studies were categorized as having a moderate risk of bias in participant selection (n= 24/46). This bias is due to patient characteristics observed after the start of the intervention. We found a low risk of bias in the classification of the intervention (n= 29/46). As most studies were observational and retrospective, no information on deviation from intervention was provided (n= 31/46), and if data were missing, they were excluded from the analysis resulting in a low risk of bias for missing data (n= 40/46). For the outcome measurement domain, 23/46 studies were classified as having a critical or serious risk of bias as in most studies the diagnosis of LLL was made by subjective measurements, or the physician made the diagnosis of LLL by clinical measurements using the ISL grading score, rather than using an objective measure such as volumetry or perimetry. The clinician already had knowledge of the intervention the patient received, which could lead to an overestimation of the diagnosis of LLL. In most studies the assessors were aware which intervention the patient had received, because of the retrospective nature of the trial. The overall risk of bias was dominated by moderate risk (n = 25/46) to serious risk (n = 14/46) due to selection bias, diagnostic bias of LLL and different follow-up times, sometimes unevenly distributed patient characteristics and a different approach to surgery and adjuvant therapy over the study period. Appendix B shows the of Risk of bias assessment for included studies using the ROBINS-I tool.

## 4 Discussion

In female patients diagnosed with gynecological cancer, the primary focus goes to treating the cancer. However, in cancer survivors the consequences of oncologic treatment have an important and often life-long impact on QoL. One of the most debilitating conditions in these cancer survivors is LLL. This study aimed to identify the main risk factors for the development of LLL following gynecological cancer treatment and to investigate its incidence. Identifying these risk factors helps to orient patient education, detection strategies and prevention. This study revealed that lymphadenectomy and radiotherapy are significant risk factors for the development of LLL in endometrial cancer patients. Additionally, some studies suggested a positive association between chemotherapy. In cervical cancer patients, radiotherapy consistently emerged as a significant risk factor for LLL, whereas chemotherapy did not show a significant association.

Risk factors have been extensively studied, and their impact is relatively clear. Several treatment-related factors, such as radiotherapy LA and the number of lymph nodes removed, influence the risk of developing LLL after gynecological cancer treatment. The most extensively studied risk factor is the number of lymph nodes removed during surgery. Although this is generally considered as the most significant risk factor, the specific cut-off value and the role of location of lymph node removal remain unclear (12, 24, 29–32, 34, 36, 37, 42, 52, 53). The impact of LA on LLL risk was only demonstrated in women with endometrial cancer. Moreover, the region most prone for developing LLL is the region of the circumflex arterial lymph nodes. Radiotherapy is described in several studies as a significant risk factor for LLL (12, 17, 23, 24, 27, 29, 32–34, 36, 37, 42, 43, 46, 49–51, 53, 57). The hypothesis is that radiotherapy may have a synergistic effect with surgery on the development of LLL (60). Surgery may directly damage the lymphatic system, disrupting lymphatic drainage, while radiotherapy may exacerbate the damage due to fibrosis and obstruction of collateral lymphatic circulation formation (54, 61). The role of other treatment-related factors was not conclusive, including type of surgery - open versus closed approach - type of hysterectomy, or application of retroperitoneal closure. No association could be demonstrated between presence of lymph node metastases and LLL (22, 28, 31, 33, 35, 41, 49, 50). Most studies also show no association between cancer type, histology or FIGO classification and lymphedema (18, 22, 28, 32, 35, 41, 49, 50, 55). Only 1 study found that lymphoedema was more common in patients with endometrial cancer than in those with ovarian cancer (5). However, there are other risk factors where the evidence remains inconclusive. These may include patient-related variables such as BMI and age. Specific patient subgroups, such as those with higher BMI or older age, may be at greater risk for developing LLL due to their overall health status and the potential for more extensive surgical interventions. Severe obesity is a recognized risk factor for secondary lymphoedema in the legs following cancer surgery (46). Studies have shown that obesity can exacerbate the risk of lymphedema by affecting lymphatic function. Further research is needed to clarify these associations and to develop targeted prevention strategies for high-risk groups. This includes understanding how BMI and age interact with other risk factors and treatment modalities to influence the risk of LLL. The role of BMI in the development of LLL is not clear. Due to significant variability in data, caution should be exercised when interpreting BMI as a risk factor for LLL. In cervical cancer, articles that demonstrated an association between BMI and lymphedema did not specifically define the BMI values but indicated a clear link between higher BMI and LLL risk (17, 24). However, the role of BMI remains equivocal, as most studies failed to find an association between BMI and LLL (7, 16–18, 27, 32, 35, 36, 39, 43, 45, 48, 51, 54). The exact mechanisms by which BMI influences lymphedema development are not fully understood. Hypotheses are that a higher BMI may lead to increased pressure on lymphatic vessels, obstructing lymph flow and impairing drainage. Obesity can alter immune function and excess adipose tissue might trigger chronic inflammation, affecting lymphatic function (62, 63). The impact of BMI on LLL risk varies across studies, and specific BMI tresholds are not well-defined. The role of age in the development of LLL post-endometrial cancer treatment remains ambiguous. While Wedin et al. suggested that younger women are at higher risk due to potentially higher activity levels leading to more damage to lymphatic vessels, other studies indicated that patients aged 60–65 years or older are more susceptible (19, 37). This discrepancy underscores the need for more targeted research to clarify age-related risks. It is important to note that the varying age cutoffs used across studies may not fully capture the impact of age on lymphedema development. In cervical cancer age was not consistently associated with cancer risk but may still play a role in LLL development. Additionally, younger women often present with more advanced cancers, requiring more invasive and hence more lympho-destructive oncologic treatment (64).

The reported incidence of LLL following gynecological cancer treatment varies significantly across studies and appears strongly influenced by the diagnostic method and threshold applied. For instance, using a diagnostic cut-off of a 5–10% increase in leg circumference may underestimate incidence particularly in patients with bilateral LLL, where asymmetry is less pronounced. In 2017, Biglia et al. reported LLL incidences of 11-24.1% for cervical cancer, 1.2-47% for endometrial cancer, 4.7-40% for ovarian cancer and 30-70% for vulvar cancer following treatment (6). Similarly Dessources et al. documented ranges from 7.4% to 58% in cervical cancer, 1.2% to 80.4% in endometrial cancer, 5.6% to 35.3% in ovarian cancer and 0% to 58.3% for vulvar/vaginal cancer (7). In our current review, incidence rates ranged from 7.8–55.9% for cervical cancer, 1.2–47% for endometrial cancer, 5.6–30.4% for ovarian cancer, and 10.1–43% for vulvar/vaginal cancer. These variations underscore the absence of a standardized diagnostic approach. Methods used across studies included patient-reported questionnaires, clinical examinations, and imaging techniques, each with different sensitivity and specificity. For example, studies relying solely on questionnaires may overestimate incidence due to subjective bias, while more objective volumetric methods although more specific might miss subtler cases, especially bilateral ones. Diagnostic methods varied widely, including questionnaires, clinical examinations, and imaging techniques. This analysis highlights how variations in diagnostic approaches can influence the reported incidence rates. For instance, studies using only questionnaires may report higher subjective incidence rates compared to those using clinical examinations or imaging, which might provide more objective measurements. The lack of a standardized diagnostic approach for LLL contributes to the wide range of reported incidence rates. Establishing a gold standard for diagnosis could help in obtaining more consistent and comparable data across studies. Our findings underscore the need for standardized diagnostic criteria in future research to reduce variability and improve the accuracy of LLL incidence reporting. Although volumetric measures are more objective, they are less sensitive and may underestimate the incidence of LLL, particularly for grade I LLL. Other complicating factors are its frequent bilateral occurrence, which hinders limb comparison, and central manifestation in the groin or pubic region, where circumference measurement is impractical. The most objective tool for objective diagnosis of impaired lymphatic drainage is lymphoscintigraphy, a time-consuming and ionizing technical exam, requiring advanced technological infrastructure. It is valuable in detecting advanced LLL, but seems less sensitive in early stage LLL or in LLL of the pelvic region (65). In many studies, diagnosis was based on scoring questionnaires to detect LLL, such as the Gynecologic Cancer Lymphedema Questionnaire (GCLQ) and the Self-Report Lower-Extremity Lymphedema Screening Questionnaire (LELSQ). Another frequent strategy is clinical assessment using the ISL grading system. Only few studies performed imaging techniques for validation of diagnosis. Circumference and volumetric measurements are more frequently used, with some studies applying a volume increase of 5%, while others use a 20% difference between both legs to define LLL (58). No metachronous evaluations were found. Additionally, the heterogeneity in treatment approaches—ranging from surgery alone, surgery with LA, surgery combined with brachytherapy, surgery with sentinel lymph node biopsy, to surgery with sentinel lymph node biopsy and adjuvant radiotherapy—adds another layer of complexity. Our meta-analysis did not find a statistically significant association between the number of lymph nodes retrieved, surgical approach (open vs. minimal invasive surgery (MIS)), and the incidence of LLL in endometrial cancer patients. This finding contrasts with several previous meta-analyses and reviews, including Desources et al., who reported that a greater number of lymph nodes removed was associated with increased risk of lymphedema (6, 7). Several factors may explain this discrepancy. First, there was substantial heterogeneity across studies in our meta-analysis, both in terms of methodology and outcome definitions. For example, the number of lymph nodes retrieved was inconsistently reported, and thresholds for “extensive” dissection varied between studies, which may have masked a potential dose-response effect. Second, data on surgical approach (open vs. MIS) were limited in the included studies and often not stratified or analyzed separately, restricting our ability to draw firm conclusions on this variable. Third, unlike some broader reviews, our meta-analysis included only studies that specifically analyzed lymphedema as an outcome in endometrial cancer and reported effect sizes (e.g., odds ratios), which may have led to the exclusion of more general studies that reported on surgical factors but did not focus on lymphedema risk. Lastly, our meta-regression did not identify a significant moderating effect of the median number of lymph nodes removed, suggesting that while lymphadenectomy overall increases risk, the extent of dissection may not be a linear predictor, or the signal may be diluted by confounding factors such as radiotherapy. These methodological and reporting differences likely contribute to the divergence from prior findings. Although sentinel lymph node (SLN) biopsy is increasingly adopted in the surgical management of endometrial cancer to reduce morbidity, our meta-analysis did not include studies that specifically evaluated LLL risk in patients who underwent SLN mapping alone. Most studies included in our review focused on patients treated with full pelvic and/or para-aortic LA, and data distinguishing SLN-only procedures were either absent or not reported separately. Given the growing clinical relevance of SLN biopsy, future prospective studies are needed to assess the incidence and risk factors of LLL in this specific subgroup. The variability in both diagnostic and treatment protocols underscore the need for standardized guidelines to accurately assess and manage LLL in gynecological cancer patients.

A limitation of our review is that most of the included studies are retrospective and vary significantly in design, population, and methodology. Only for endometrial and cervical cancer were the data sufficient and consistent enough to allow pooling for meta-analysis. The large variability between studies in type of cancer, diagnostic strategy, population size, and trial methodology may introduce bias and limit the ability to draw consistent conclusions. Effectively, there is no consensus on the diagnosis of LLL, with different studies using various criteria and methods for diagnosis, hence undermining the comparability of the results. Not all studies investigated the same risk factors, and those that did often used different definitions and thresholds. Additionally, most of the included studies are from Asia, which may not be directly applicable to Western populations due to potential cultural differences. Another difficulty is the variation in therapeutic approaches between regions and over time. Another difficulty is the variation in therapeutic approaches between regions and over time. Specifically, data on SLN biopsy alone were limited or not reported separately in most studies, making it difficult to assess the risk of LLL in patients undergoing SLN mapping without full LA. This represents an important gap, especially given the increasing adoption of SLN in clinical practice. Moreover, the presence of moderate statistical heterogeneity indicates variability in the effect estimates across studies. Clinical heterogeneity, arising from variations in patient characteristics, disease stages, and treatment protocols, complicates the pooling of data. This inherent variability among studies remains a significant limitation. We also combined patients with early-stage (I-II) and advanced-stage (III) disease, as well as those with and without lymph node dissection and involvement. Although subgroup analyses supported the pooling of these groups, the potential differences in baseline risks for lymphedema may introduce bias.

LA and radiotherapy remain the most clearly established risk factors for LLL following treatment for gynecological cancers. However, the evidence for other factors such as BMI, age, and chemotherapy is inconsistent and often confounded by methodological variability. The wide variation in diagnostic criteria and treatment protocols further complicates comparison across studies. Notably, data on SLN biopsy—a procedure increasingly used to minimize morbidity—are scarce, underscoring an important gap in the current literature. Our findings highlight the need for future prospective studies with standardized diagnostic tools, consistent definitions of risk factors, and focused analyses on emerging treatment modalities such as SLN. Ultimately, improving consistency in study design and reporting is essential for developing targeted prevention strategies and improving long-term outcomes for cancer survivors.

## 5 Conclusion

This systematic review confirms that LA, particularly involving the circumflex arterial lymph nodes and radiotherapy are the most consistently reported treatment-related risk factors for the development of LLL in gynecological cancer patients, especially in those with endometrial and cervical cancer. In contrast, the role of other variables such as BMI, age, cancer histology, and surgical approach remains inconclusive due to inconsistent study designs, heterogeneous reporting, and lack of standardized definitions. A major challenge in interpreting LLL incidence across studies lies in the absence of a clear diagnostic standard. The variability in diagnostic tools, thresholds for defining LLL, and methods of assessment has led to a wide range of reported incidence rates across tumor types. This diagnostic heterogeneity significantly limits comparability and hinders the ability to draw firm conclusions. Our meta-analysis did not identify a significant association between the number of lymph nodes retrieved or surgical approach (open vs. MIS) and the incidence of LLL, which may reflect methodological limitations, variability in reporting, and the exclusion of studies not specifically focused on lymphedema. Moreover, the lack of data on patients treated solely with SLN biopsy represents a critical gap in the literature, particularly as SLN mapping gains prominence in clinical practice. Despite these limitations, this review represents the most comprehensive synthesis of current evidence on LLL risk to date. It underscores the need for standardized diagnostic tools, clearer treatment variable definitions, and inclusion of both early and advanced disease stages in future prospective trials. Improved understanding of how risk factors both treatment-related and patient-specific interact will be essential for developing targeted prevention and management strategies for high-risk subgroups. Ultimately, such efforts will enhance clinical decision-making and improve outcomes for gynecologic cancer survivors.

## Data Availability

The original contributions presented in the study are included in the article/**Supplementary Material**. Further inquiries can be directed to the corresponding author.
