# Supplementary material for: Risk factors for lower limb lymphedema after gynecological cancer treatment: a systematic review
Source: Front Oncol. 2025 May 20;15:1561836. doi: 10.3389/fonc.2025.1561836 (PMC12129805; doi:10.3389/fonc.2025.1561836)
Supplement: Supplementary file 1 [file DataSheet1.docx]

Supplementary Material

# Supplementary Data

**Appendix 1 :** Search Strategy : Risk factors for lymphedema of the lower limb following treatment for gynecological cancer : a systematic review of the literature

**PubMed**

(“Lymphedema” [MeSH] OR “lymphedema” [TIAB] OR “lymphoedema” [TIAB] OR “lymphatic edema” [TIAB] OR “lymphatic oedema”[TIAB] OR “lymph edema”[TIAB] OR “lymph oedema”[TIAB] OR “lymphooedema”[TIAB] OR “lymphostatic oedema”[TIAB] OR “leg oedema”[TIAB] OR “leg edema”[TIAB] OR “lymphedemas”[TIAB] OR “lymphoedemas”[TIAB])

AND

(“genital neoplasms, female”[MeSH] OR “gynecologic neoplasms”[TIAB] OR “ovarian neoplasms”[MeSH] OR “ovarian neoplasms”[TIAB] OR “ovarian cancer”[TIAB] OR “ovary cancer”[TIAB] OR “ovary neoplasms”[TIAB] OR “cancer of ovary”[TIAB] OR “cancer of the ovary”[TIAB] OR “ovarian tumor” [TIAB] OR “ovarian tumour”[TIAB] OR “ovary tumor”[TIAB] OR “ovary tumor”[TIAB] OR “vulvar neoplasms”[MeSH] OR “vulvar neoplasms”[TIAB] OR “vulvar cancer”[TIAB] OR “vulva neoplasms”[TIAB] OR “vulva cancer”[TIAB] OR “cancer of vulva”[TIAB] OR “cancer of the vulva”[TIAB] OR “vulvar tumor”[TIAB] OR “vulvar tumour”[TIAB] OR “tumor of the vulva”[TIAB] OR “vulva tumor”[TIAB] OR “vulva tumour”[TIAB] OR “endometrial neoplasms”[MeSH] OR “endometrial neoplasms”[TIAB] OR “cancer of endometrium”[TIAB] OR “endometrial cancer”[TIAB] OR “endometrial carcinoma”[TIAB] OR “endometrium cancer”[TIAB] OR “endometrial tumor”[TIAB] OR “endometrial tumour”[TIAB] OR “endometrium tumor”[TIAB] OR “uterine neoplasms”[MeSH] OR “uterine neoplasms”[TIAB] OR “cancer of uterus”[TIAB] OR “neoplasms uterus”[TIAB] OR “uterus cancer”[TIAB] OR “uterus neoplasms”[TIAB] OR “uterine tumor”[TIAB] OR “uterine tumour”[TIAB] OR “uterus tumor”[TIAB] OR “uterus tumour”[TIAB] OR “pelvic neoplasms”[MeSH] OR “pelvic neoplasms”[TIAB] OR “pelvic cancer”[TIAB] OR “pelvic tumor”[TIAB] OR “pelvic tumour”[TIAB])

AND

(“general surgery”[MeSH] OR ”surgery”[TIAB] OR “gynecologic surgical procedures”[MeSH] OR “adnexa uteri/surgery”[MeSH] OR “uterus/surgery”[MeSH] OR gynecolog*[TIAB] OR gynaecolg*[TIAB] OR lavh[TIAB] OR lash[TIAB] OR lsh[TIAB] OR(uter*[TIAB] AND extirpation[TIAB]) OR hysterectom*[TIAB] OR adnexectom*[TIAB] OR ovariectom*[TIAB] OR (adnex*[TIAB] AND (extirpation[TIAB] OR surgery[TIAB] OR surgical[TIAB])) OR salpingectom*[TIAB] OR salingoo*[TIAB] OR (salpingo[TIAB] AND (oophorectom*[TIAB] OR ophorectom*[TIAB])) OR “radiotherapy”[MeSH] OR “radiotherapy”[TIAB] OR “radiation therapy”[TIAB] OR “radiation treatment”[TIAB] OR “radiotherapy”[subheading] OR “radiosurgery”[MeSH] OR radiotherap*[TIAB] OR radiati*[TIAB] OR radiosurg*[TIAB] OR irradiati*[TIAB] OR “x ray therapy”[TIAB] OR “x ray therapies”[TIAB] OR radioimmunotherap*[TIAB] OR immunoradiotherap*[TIAB])

AND

(“risk factors”[MeSH] OR “risk factors”[TIAB] OR “risk factor”[TIAB] OR “complication”[TIAB] OR “complications”[TIAB] OR “ long term effect”[TIAB] OR “long term effects”[TIAB] OR “late effect”[TIAB] OR “late effects”[TIAB])

**Embase**

‘lymphedema’/exp OR ‘lymphedema’:ab,ti OR ‘lymphoedema’:ab,ti OR ‘lymph edema’:ab,ti OR ‘lymph oedema’:ab,ti OR ‘lymphatic edema’:ab,ti OR ‘lymphatic oedema’:ab,ti OR ‘lymphooedema’:ab,ti OR ‘lymphostatic oedema’:ab,ti OR ‘lymphostatic edema’:ab,ti OR ‘leg oedema’:ab,ti OR ‘leg edema’:ab,ti OR ‘lymphedemas’:ab,ti OR ‘lymphoedemas’:ab,ti

AND

‘female genital tract tumor’/exp OR ‘female genital tract tumor’:ab,ti OR ‘female genital tract tumour’:ab,ti OR ‘female genital neoplasms’:ab,ti OR ‘gynecologic neoplasm’:ab,ti OR ‘gynecologic neoplasms’:ab,ti OR ‘gynecologic cancer’:ab,ti OR ‘gynecologic cancers’:ab,ti OR ‘gynecologic tumor’:ab,ti OR ‘gynecologic tumors’:ab,ti OR ‘gynecologic tumour’:ab,ti OR ‘gynecologic tumours’:ab,ti OR ‘gynecologic carcinoma’:ab,ti OR ‘gynecological neoplasm’:ab,ti OR ‘gynecological neoplasms’:ab,ti OR ‘gynecological cancer’:ab,ti OR ‘gynecological cancers’:ab,ti OR ‘gynecological tumor’:ab,ti OR ‘gynecological tumors’:ab,ti OR ‘gynecological tumour’:ab,ti OR ‘gynecological tumours’:ab,ti OR ‘gynecological carcinoma’:ab,ti OR ‘gynaecological neoplasm’:ab,ti OR ‘gynaecological neoplasms’:ab,ti OR ‘gynaecological cancer’:ab,ti OR ‘gynaecological cancers’:ab,ti OR ‘gynaecological tumor’:ab,ti OR ‘gynaecological tumors’:ab,ti OR ‘gynaecological tumour’:ab,ti OR ‘gynaecological tumours’:ab,ti OR ‘gynaecological carcinoma’:ab,ti OR ‘gynaecologic neoplasm’:ab,ti OR ‘gynaecologic neoplasms’:ab,ti OR ‘gynaecologic cancer’:ab,ti OR ‘gynaecologic cancers’:ab,ti OR ‘gynaecologic tumor’:ab,ti OR ‘gynaecologic tumors’:ab,ti OR ‘gynaecologic tumour’:ab,ti OR ‘gynaecologic tumours’:ab,ti OR ‘gynaecologic carcinoma’:ab,ti OR ‘female genital system tumour’:ab,ti OR ‘female genital system tumor’:ab,ti

OR ‘ovary tumor’/exp OR ‘ovarian neoplasm’:ab,ti OR ‘ovarian neoplasms’:ab,ti OR ‘ovarian cancer’:ab,ti OR ‘ovarian cancers’:ab,ti OR ‘ovarian tumor’:ab,ti OR ‘ovarian tumors’:ab,ti OR ‘ovarian tumour’:ab,ti OR ‘ovarian tumours’:ab,ti OR ‘ovarian carcinoma’:ab,ti OR ‘ovary neoplasm’:ab,ti OR ‘ovary neoplasms’:ab,ti OR ‘ovary cancer’:ab,ti OR ‘ovary cancers’:ab,ti OR ‘ovary tumor’:ab,ti OR ‘ovary tumors’:ab,ti OR ‘ovary tumour’:ab,ti OR ‘ovary tumours’:ab,ti OR ‘ovary carcinoma’:ab,ti OR ‘cancer of ovary’:ab,ti OR ‘cancer of the ovary’:ab,ti

OR ‘vulva tumor’/exp OR ‘vulvar neoplasm’:ab,ti OR ‘vulvar neoplasms’:ab,ti OR ‘vulvar cancer’:ab,ti OR ‘vulvar tumor’:ab,ti OR ‘vulvar tumors’:ab,ti OR ‘vulvar tumour’:ab,ti OR ‘vulvar tumours’:ab,ti OR ‘vulvar carcinoma’:ab,ti OR ‘tumor of the vulva’:ab,ti OR ‘tumour of the vulva’:ab,ti OR ‘vulva neoplasm’:ab,ti OR ‘vulva neoplasms’:ab,ti OR ‘vulva cancer’:ab,ti OR ‘vulva tumor’:ab,ti OR ‘vulva tumors’:ab,ti OR ‘vulva tumour’:ab,ti OR ‘vulva tumours’:ab,ti OR ‘vulva carcinoma’:ab,ti OR ‘cancer of vulva’:ab,ti OR ‘cancer of the vulva’:ab,ti

OR ‘endometrium tumor’/exp OR ‘endometrial neoplasm’:ab,ti OR ‘endometrial neoplasms’:ab,ti OR ‘endometrial cancer’:ab,ti OR ‘endometrial cancers’:ab,ti OR ‘endometrial carcinoma’:ab,ti OR ‘endometrial tumor’:ab,ti OR ‘endometrial tumors’:ab,ti OR ‘endometrial tumour’:ab,ti OR ‘endometrial tumours’:ab,ti OR ‘endometrium neoplasm’:ab,ti OR ‘endometrium neoplasms’:ab,ti OR ‘endometrium cancer’:ab,ti OR ‘endometrium cancers’:ab,ti OR ‘endometrium tumor’:ab,ti OR ‘endometrium tumors’:ab,ti OR ‘endometrium tumour’:ab,ti OR ‘endometrium tumours’:ab,ti OR ‘endometrium carcinoma’:ab,ti

OR ‘uterus cancer’:exp OR ‘uterine neoplasm’:ab,ti OR ‘uterine neoplasms’:ab,ti OR ‘uterus cancer’:ab,ti OR ‘uterine cancers’:ab,ti OR ‘uterine tumor’:ab,ti OR ‘uterine tumors’:ab,ti OR ‘uterine tumour’:ab,ti OR ‘uterine tumours’:ab,ti OR ‘uterine carcinoma’:ab,ti OR ‘cancer of uterus’:ab,ti OR ‘uterus neoplasm’:ab,ti OR ‘uterus neoplasms’:ab,ti OR ‘uterus cancer’:ab,ti OR ‘uterus cancers’:ab,ti OR ‘uterus tumor’:ab,ti OR ’uterus tumors’:ab,ti OR ‘uterus tumour’:ab,ti OR ‘uterus tumours’:ab,ti OR ‘uterus carcinoma’:ab,ti

OR ‘pelvic neoplasm’:ab,ti OR ‘pelvic neoplasms’:ab,ti OR ‘pelvic cancer’:ab,ti OR ‘pelvic cancers’:ab,ti OR ‘pelvic tumor’:ab,ti OR ’pelvic tumors’:ab,ti OR ‘pelvic tumour’:ab,ti OR ‘pelvic tumours’:ab,ti OR ‘pelvic carcinoma’:ab,ti

AND

‘gynecologic surgery’/exp OR gynecolog*:ab,ti OR gynaecolog*:ab,ti OR lavh:ab,ti OR lash:ab,ti OR lsh:ab,ti OR (uter*:ab,ti AND extirpation:ab,ti) OR hysterectom*:ab,ti OR adnexectom*:ab,ti OR ovariectom*:ab,ti OR (adnex*:ab,ti AND (extirpation:ab,ti OR surgery:ab,ti OR surgical:ab,ti)) OR salpingectom*:ab,ti OR salpingoo*:ab,ti OR (salpingo:ab,ti AND (oophorectom*:ab,ti OR ophorectom*:ab,ti))

OR

'radiotherapy'/exp OR ‘radiotherapy’:ab,ti OR ‘radiation therapy’:ab,ti OR ‘radiation treatment’:ab,ti

AND

'risk factor'/exp OR ‘risk factor’:ab,ti OR ‘risk factors’:ab,ti OR ‘complication’:ab,ti OR ‘complications’:ab,ti OR ‘long term effect’:ab,ti OR ‘long term effects’:ab,ti OR ‘late effect’:ab,ti OR ‘late effects’:ab,ti

**CENTRAL**

[mh “Lymphedema”] OR “lymphedema”:ti,ab,kw OR “lymphoedema”:ti,ab,kw OR “lymphatic edema”:ti,ab,kw OR “lymphatic oedema”:ti,ab,kw OR “lymph edema”:ti,ab,kw OR “lymph oedema”:ti,ab,kw OR “lymphooedema”:ti,ab,kw OR “lymphedema”:ti,ab,kw  OR “lymphostatic oedema”:ti,ab,kw OR “lymphostatic edema”:ti,ab,kw OR “leg oedema”:ti,ab,kw OR “leg edema”:ti,ab,kw OR “lymphedemas”:ti,ab,kw OR “lymphoedemas”:ti,ab,kw

AND

[mh “genital neoplasms, female”] OR “gynecologic neoplasm”:ti,ab,kw OR “gynecologic neoplasms”:ti,ab,kw OR “gynecologic cancer”:ti,ab,kw OR “gynecologic cancers”:ti,ab,kw OR “gynecologic tumor”:ti,ab,kw OR “gynecologic tumors”:ti,ab,kw OR “gynecologic tumour”:ti,ab,kw OR “gynecologic tumours”:ti,ab,kw OR “gynecologic carcinoma”:ti,ab,kw or “gynecological neoplasm”:ti,ab,kw OR “gynecologic neoplasms”:ti,ab,kw OR “gynecological cancer”:ti,ab,kw OR “gynecological cancers”:ti,ab,kw OR “gynecological tumor”:ti,ab,kw or “gynecological tumour”:ti,ab,kw OR “gynecological tumours”:ti,ab,kw OR “gynecological carcinoma”:ti,ab,kw OR “gynaecologic neoplasm”:ti,ab,kw OR “gynaecologic neoplasms”:ti,ab,kw OR ”gynaecologic cancer”:ti,ab,kw OR “gynaecologic cancers”:ti,ab,kw OR “gynaecologic tumor”:ti,ab,kw OR “gynaecologic tumors”:ti,ab,kw OR “gynaecologic tumour”:ti,ab,kw OR “gynaecologic tumours”:ti,ab,kw OR “gynaecologic carcinoma”:ti,ab,kw OR “gynaecological neoplasm”:ti,ab,kw OR “gynaecological neoplasms”:ti,ab,kw OR “gynaecological cancer”:ti,ab,kw OR “gynaecological cancers”:ti,ab,kw OR “gynaecological tumor”:ti,ab,kw OR “gynaecological tumors”:ti,ab,kw OR “gynaecological tumour”:ti,ab,kw OR “gynaecological tumours”:ti,ab,kw OR “gynaecological carcinoma”:ti,ab,kw OR ((“genital neoplasm”:ti,ab,kw OR “genital neoplasms”:ti,ab,kw OR “genital cancer”:ti,ab,kw OR “genital cancers”:ti,ab,kw OR “genital tumor”:ti,ab,kw OR “genital tumors”:ti,ab,kw OR “genital tumour”:ti,ab,kw OR “genital tumours”:ti,ab,kw OR “genital carcinoma”:ti,ab,kw) AND ([mh “female”] OR [mh “women”] OR female*:ti,ab,kw OR woman:ti,ab,kw or women:ti,ab,kw OR girl:ti,ab,kw OR girls:ti,ab,kw OR feminine*:ti,ab,kw))

OR [mh “ovarian neoplasms”] OR “ovarian neoplasm”:ti,ab,kw OR “ovarian neoplasms”:ti,ab,kw OR “ovarian cancer”:ti,ab,kw OR “ovarian cancers”:ti,ab,kw OR “ovarian tumor”:ti,ab,kw OR “ovarian tumors”:ti,ab,kw OR “ovarian tumour”:ti,ab,kw OR “ovarian tumours”:ti,ab,kw OR “ovarian carcinoma”:ti,ab,kw OR “ovary neoplasm”:ti,ab,kw OR “ovary neoplasms”:ti,ab,kw OR “ovary cancer”:ti,ab,kw OR “ovary cancers”:ti,ab,kw OR “ovary tumor”:ti,ab,kw OR “ovary tumors”:ti,ab,kw OR “ovary tumour”:ti,ab,kw OR “ovary tumours”:ti,ab,kw OR “ovary carcinoma”:ti,ab,kw OR “cancer of ovary”:ti,ab,kw OR “cancer of the ovary”:ti,ab,kw

OR [mh “vulvar neoplasms”] OR “vulvar neoplasm”:ti,ab,kw OR “vulvar neoplasms”:ti,ab,kw OR “vulvar cancer”:ti,ab,kw OR “vulvar cancers”:ti,ab,kw OR “vulvar tumor”:ti,ab,kw OR “vulvar tumors”:ti,ab,kw OR “vulvar tumour”:ti,ab,kw OR “vulvar tumours”:ti,ab,kw OR “vulvar carcinoma”:ti,ab,kw OR “vulva neoplasm”:ti,ab,kw OR “vulva neoplasms”:ti,ab,kw OR “vulva cancer”:ti,ab,kw OR “vulva cancers”:ti,ab,kw OR “vulva tumor”:ti,ab,kw OR “vulva tumors”:ti,ab,kw OR “vulva tumour”:ti,ab,kw OR “vulva tumours”:ti,ab,kw OR “vulva carcinoma”:ti,ab,kw

OR [mh “endometrial neoplasms”] OR “endometrial neoplasm”:ti,ab,kw OR “endometrial neoplasms”:ti,ab,kw OR “endometrial cancer”:ti,ab,kw OR “endometrial cancers”:ti,ab,kw OR “endometrial tumor”:ti,ab,kw OR “endometrial tumours”:ti,ab,kw OR “endometrial tumour”:ti,ab,kw OR “endometrial tumours”:ti,ab,kw OR “endometrial carcinoma”:ti,ab,kw OR “endometrium neoplasm”:ti,ab,kw OR “endometrium neoplasms”:ti,ab,kw OR “endometrium cancer”:ti,ab,kw OR “endometrium cancers”:ti,ab,kw OR “endometrium tumor”:ti,ab,kw OR “endometrium tumors”:ti,ab,kw OR “endometrium tumour”:ti,ab,kw OR “endometrium tumours”:ti,ab,kw OR “endometrium carcinoma”:ti,ab,kw

OR [mh “uterine neoplasms”] OR “uterine neoplasm”:ti,ab,kw OR “uterine neoplasms”:ti,ab,kw OR “uterine cancer”:ti,ab,kw OR “uterine cancers”:ti,ab,kw OR “uterine tumor”:ti,ab,kw OR “uterine tumors”:ti,ab,kw OR “uterine tumour”:ti,ab,kw OR “uterine tumours”:ti,ab,kw OR “uterine carcinoma”:ti,ab,kw OR “cancer of uterus”:ti,ab,kw OR “uterus neoplasm”:ti,ab,kw OR “ uterus neoplasms”:ti,ab,kw OR “uterus cancer”:ti,ab,kw OR “uterus tumor”:ti,ab,kw OR “uterus tumors”:ti,ab,kw OR “uterus tumour”:ti,ab,kw OR “uterus tumours”:ti,ab,kw OR “uterus carcinoma”:ti,ab,kw

OR [mh “pelvic neoplasms”] OR “pelvic neoplasm”:ti,ab,kw OR “pelvic neoplasms”:ti,ab,kw OR “pelvic cancer”:ti,ab,kw OR “pelvic cancers”:ti,ab,kw OR “pelvic tumor”:ti,ab,kw OR “pelvic tumors”:ti,ab,kw OR “pelvic tumour”:ti,ab,kw OR “pelvic tumours”:ti,ab,kw OR “pelvic carcinoma”:ti,ab,kw

AND

[mh “general surgery”] OR ”surgery”:ti,ab,kw OR [mh “gynecologic surgical procedures”] OR [mh “adnexa uteri/surgery”] OR [mh “uterus/SU”] OR gynecolog*:ti,ab,kw OR gynaecolg*:ti,ab,kw OR lavh:ti,ab,kw OR lash:ti,ab,kw OR lsh:ti,ab,kw OR (uter*:ti,ab,kw AND extirpation:ti,ab,kw) OR hysterectom*:ti,ab,kw OR adnexectom*:ti,ab,kw OR ovariectom*:ti,ab,kw OR (adnex*:ti,ab,kw AND (extirpation:ti,ab,kw OR surgery:ti,ab,kw OR surgical:ti,ab,kw)) OR salpingectom*:ti,ab,kw OR salingoo*:ti,ab,kw OR (salpingo:ti,ab,kw AND (oophorectom*:ti,ab,kw OR ophorectom*:ti,ab,kw)) OR [mh “radiotherapy”] OR “radiotherapy”:ti,ab,kw OR “radiation therapy”:ti,ab,kw OR “radiation treatment”:ti,ab,kw OR [mh “radiosurgery”] OR radiotherap*:ti,ab,kw OR radiati*:ti,ab,kw OR radiosurg*:ti,ab,kw OR irradiati*:ti,ab,kw OR “x ray therapy”:ti,ab,kw OR “x ray therapies”:ti,ab,kw OR radioimmunotherap*:ti,ab,kw OR immunoradiotherap*:ti,ab,kw

AND

[mh “risk factors”] OR “risk factors”:ti,ab,kw OR “risk factor”:ti,ab,kw OR “complication”:ti,ab,kw OR “complications”:ti,ab,kw OR “long term effect”:ti,ab,kw OR “long term effects”:ti,ab,kw OR “late effect”:ti,ab,kw OR “late effects”:ti,ab,kw OR “lymph node dissection”:ti,ab,kw OR “adipose tissue”:ti,ab,kw OR “fatty tissue”:ti,ab,kw OR “fat tissue”:ti,ab,kw
