# Supplementary material for: Risk factors for lower limb lymphedema after gynecological cancer treatment: a systematic review
Source: Front Oncol. 2025 May 20;15:1561836. doi: 10.3389/fonc.2025.1561836 (PMC12129805; doi:10.3389/fonc.2025.1561836)
Supplement: Supplementary file 2 [file Table1.docx]

Supplementary Material

| Study | Confounding | Selection | Classification | Deviation | Missing Data | Measurement | reported result | Overall |
| --- | --- | --- | --- | --- | --- | --- | --- | --- |
| Yost et al., 2014, USA (8) | Low | Low | NI | NI | Low | Serious | Low | Low |
| Yoshihara et al., Japan, 2020 (9) | Moderate | Low | Low | NI | NI | Low | Low | Low |
| Yamazaki et al., Japan, 2015 (10) | Serious | Moderate | Low | NI | Low | Moderate | Low | Moderate |
| Wong et al., United Kingdom, 2021 (11) | Moderate | Serious | Moderate | Moderate | Low | Serious | Low | Serious |
| Wedin et al., Sweden, 2021 (12) | Moderate | Low | Low | Low | Low | Serious | Moderate | Moderate |
| Wedin et al., Sweden, 2020 (13) | Serious | Low | Moderate | Serious | Low | Serious | Low | Moderate |
| Walker et al., United Kingdom, 2011 (14) | Moderate | Moderate | Low | NI | Low | Serious | Critical | Moderate |
| Volpi et al., Italy, 2019 (15) | Low | Low | Low | NI | Low | Serious | Moderate | Moderate |
| Togami et al., Japan, 2020 (16) | Moderate | Moderate | Moderate | Moderate | Low | Serious | Low | Moderate |
| Togami et al., Japan, 2020 (17) | Moderate | Moderate | Moderate | NI | Low | Serious | Low | Moderate |
| Togami et al., Japan, 2018 (18) | Moderate | Moderate | Serious | Low | Low | Serious | Low | Serious |
| Todo et al., Japan, 2015 (19) | Serious | Serious | Moderate | Moderate | Low | Moderate | Moderate | Serious |
| Todo et al., Japan, 2010 (20) | Moderate | Serious | Moderate | Low | Low | Moderate | Serious | Moderate |
| Tada et al., Japan, 2009 (4) | Serious | Moderate | Low | NI | Low | Critical | Serious | Serious |
| Kong et al., Korea, 2021 (21) | Serious | Moderate | Low | Moderate | Low | NI | Low | Low |
| Ryan et al., 2003, Australia (22) | Moderate | Moderate | Low | NI | Low | Serious | Low | Moderate |
| Rebegea et al., 2020 Romania (23) | Moderate | Moderate | Low | Moderate | Low | Moderate | Low | Serious |
| Pigot et al., 2020, Australia (24) | Low | Low | Low | Low | Moderate | Moderate | Moderate | Moderate |
| Onoda et al., 2016, Japan (25) | Serious | Moderate | Low | Low | Low | Moderate | Low | Moderate |
| Ohba et al., 2011, Japan (26) | Low | Moderate | Low | Low | Low | Moderate | Low | Moderate |
| Nakamura et al., 2016, Japan (27) | Serious | Moderate | Low | NI | Low | Low | Low | Moderate |
| Mitra et al., 2016, USA (28) | Low | Moderate | NI | Low | Low | Critical | Critical | Serious |
| Liu et al., 2022, China (29) | Low | Moderate | Moderate | NI | Low | Moderate | Low | Moderate |
| Bae et al., 2016, Korea (30) | Low | Moderate | Serious | NI | Low | Critical | Low | Serious |
| Lee et al., 2021, Korea (31) | Moderate | Serious | Serious | NI | Low | Moderate | Low | Serious |
| Kuroda et al., 2017, Japan (32) | Low | Moderate | Serious | NI | Low | Critical | Moderate | Moderate |
| Kunitake et al., 2020, Japan (33) | Low | Moderate | Low | NI | Serious | Serious | Moderate | Serious |
| Kondo et al., 2013, Japan (34) | Low | Low | Low | NI | Low | Low | Low | Low |
| Kizer et al., 2011, USA (35) | Low | Moderate | Low | NI | Moderate | Moderate | Low | Moderate |
| Kim et al., 2017, Korea (36) | Low | Moderate | Low | NI | Low | Low | Low | Moderate |
| Kim et al., 2012, Korea (37) | Low | Low | Low | NI | Low | Moderate | Moderate | Moderate |
| Ki et al., 2016, Korea (38) | Low | Low | Low | NI | Low | Low | Low | Low |
| Kasuya et al., 2011, Japan (39) | Moderate | Serious | Serious | NI | Low | Moderate | Low | Moderate |
| Chang et al., 2019, Korea (40) | Low | Moderate | Low | NI | Low | Low | Moderate | Moderate |
| Hoogendam et al., 2014, Netherlands (41) | Moderate | Moderate | Low | NI | Low | Moderate | Low | Moderate |
| Hong et al., 2002, Taiwan (42) | Low | Moderate | Low | NI | Low | Serious | Low | Moderate |
| Hayes et al., 2017, Australia (43) | Low | Low | Serious | NI | Moderate | Low | Low | Moderate |
| Hareyama et al., 2011, Japan (44) | Moderate | Moderate | Low | Low | Low | Serious | Low | Moderate |
| Hareyama et al., 2015, Japan (45) | Low | Moderate | Moderate | NI | Low | Serious | Low | Moderate |
| Füller et al., 2008, Germany (46) | Serious | Moderate | Low | NI | Low | Critical | Moderate | Moderate |
| Deura et al., 2014, Japan (47) | Moderate | Moderate | Low | NI | Low | Low | Low | Low |
| Clark et al., 2016, USA (48) | Serious | Moderate | Low | NI | Low | Serious | Moderate | Moderate |
| Cirik et al., 2015, Turkey (49) | Low | Low | Low | Low | Low | Critical | Moderate | Moderate |
| Cibula et al., 2021, Switzerland (50) | Low | Low | Low | NI | Low | Low | Low | Moderate |
| Carlson et al., 2020, USA (51) | Low | Low | Low | Critical | Moderate | Serious | Moderate | Moderate |
| Biglia et al., 2015, Switzerland (52) | Serious | Moderate | Low | NI | Low | Serious | Low | Serious |
| Berger et al., 2015, USA (53) | Moderate | Moderate | Low | NI | Low | Critical | Serious | Serious |
| Beesley et al., 2015, Australia (54) | Moderate | Moderate | Low | NI | Low | Critical | Moderate | Serious |
| Balaya et al., 2018, France (55) | Low | Moderate | Serious | NI | Moderate | NI | Serious | Serious |
| Achouri et al., 2013, France (56) | Serious | Low | Serious | NI | Moderate | Moderate | Low | Serious |
| Abu-Rustum et al., 2006, USA (57) | Low | Moderate | Low | Serious | Low | Moderate | Low | Moderate |

NI : No information

Appendix 2 : Table Risk of bias assessment for included studies using the Risk of Bias in Non-Randomized Studies of Interventions (ROBINS-I) tool
